# Assessment of dietary diversity and nutritional support for children living with HIV in the IeDEA pediatric West African cohort: a non-comparative, feasibility study

**DOI:** 10.1186/s40795-021-00486-4

**Published:** 2021-12-14

**Authors:** Julie Jesson, Ayoko Ephoevi-Ga, Marie-Hélène Aké-Assi, Sikiratou Koumakpai, Sylvie N’Gbeche, Evelyne Dainguy, Karen Malateste, Hugo Carrié, Marcelline D’Almeida, François Tanoh Eboua, Elom Takassi, Madeleine Amorissani-Folquet, Valériane Leroy, for the IeDEA West African Pediatric Collaboration, Marcel Djimon Zannou, Armel Poda, Fred Stephen Sarfo, Eugene Messou, Henri Chenal, Kla Albert Minga, Emmanuel Bissagnene, Aristophane Tanon, Moussa Seydi, Akessiwe Akouda Patassi, Sikiratou Adouni Koumakpai-Adeothy, Lorna Awo Renner, Sylvie Marie N’Gbeche, Clarisse Amani Bosse, Kouadio Kouakou, Madeleine Amorissani Folquet, François Tanoh Eboua, Fatoumata Dicko, Elom Takassi, François Dabis, Renaud Becquet, Charlotte Bernard, Shino Chassagne Arikawa, Antoine Jaquet, Karen Malateste, Elodie Rabourdin, Thierry Tiendrebeogo, Désiré Dahourou, Sophie Desmonde, Julie Jesson, Valeriane Leroy, Didier Koumavi Ekouevi, Jean-Claude Azani, Patrick Coffie, Abdoulaye Cissé, Guy Gnepa, Apollinaire Horo, Christian Kouadio, Boris Tchounga

**Affiliations:** 1 Faculté de Médecine Purpan, Inserm U1027, Université Paul Sabatier Toulouse 3, 37 Allées Jules Guesde, 31073 Toulouse Cedex 7, France; 2 CHU Sylvanus Olympio, Université de Lomé, Lomé, Togo; 3 CHU Yopougon, Service Pédiatrie, Abidjan, Côte d’Ivoire; 4 CNHU Hubert K. Maga, Cotonou, Bénin; 5 CePReF, Abidjan, Côte d’Ivoire; 6 CHU Cocody, Service Pédiatrie, Abidjan, Côte d’Ivoire; 7 University of Bordeaux, Inserm, Institut de Recherche pour le Développement (IRD), UMR 1219, Bordeaux, France

**Keywords:** Malnutrition, Children, Nutritional support, HIV, Cohort, West Africa

## Abstract

**Background:**

Nutritional care is not optimally integrated into pediatric HIV care in sub-Saharan Africa. We assessed the 6-month effect of a nutritional support provided to children living with HIV, followed in a multicentric cohort in West Africa.

**Methods:**

In 2014-2016, a nutritional intervention was carried out for children living with HIV, aged under 10 years, receiving antiretroviral therapy (ART) or not, in five HIV pediatric cohorts, in Benin, Togo and Côte d’Ivoire. Weight deficiency was assessed using two definitions: wasting (Weight for Height Z-score [WHZ] for children<5 years old or Body-Mass-Index for Age [BAZ] for ≥5 years) and underweight (Weight for Age Z-score [WAZ]) (WHO child growth standards). Combining these indicators, three categories of nutritional support were defined: 1/ children with severe malnutrition (WAZ and/or WHZ/BAZ <-3 Standard Deviations [SD]) were supported with Ready-To-Use Therapeutic Food (RUTF), 2/ those with moderate malnutrition (WAZ and/or WHZ/BAZ = [-3;-2[ SD) were supported with fortified blended flours produced locally in each country, 3/ those non malnourished (WAZ and WHZ/BAZ ≥-2 SD) received nutritional counselling only. Children were followed monthly over 6 months. Dietary Diversity Score (DDS) using a 24h recall was measured at the first and last visit of the intervention.

**Results:**

Overall, 326 children were included, 48% were girls. At baseline, 66% were aged 5-10 years, 91% were on ART, and 17% were severely immunodeficient (CD4 <250 cells/mL or CD4%<15). Twenty-nine (9%) were severely malnourished, 63 (19%) moderately malnourished and 234 (72%) non-malnourished. After 6 months, 9/29 (31%) and 31/63 (48%) recovered from severe and moderate malnutrition respectively. The median DDS was 8 (IQR 7-9) in Côte d’Ivoire and Togo, 6 (IQR 6-7) in Benin. Mean DDS was 4.3/9 (sd 1.2) at first visit, with a lower score in Benin, but with no difference between first and last visit (*p*=0.907), nor by intervention groups (*p*-value=0.767).

**Conclusions:**

This intervention had a limited effect on nutritional recovery and dietary diversity improvement. Questions remain on determining appropriate nutritional products, in terms of adherence, proper use for families and adequate energy needs coverage for children living with HIV.

**Trial registration:**

PACTR202001816232398, June 01, 2020, retrospectively registered.

**Supplementary Information:**

The online version contains supplementary material available at 10.1186/s40795-021-00486-4.

## Background

In 2020, an estimate of 1.7 million children aged less than 15 years of age were living with HIV worldwide, with more than 90% of them in sub-Saharan Africa [1]. Despite improvements in access to antiretroviral therapy (ART) and prevention from mother-to-child transmission (PMTCT), 160 000 children were newly infected with HIV [1]. With a rate of 20% of mother-to-child transmission (MTCT), the West and Central Africa region contributes to one third of these infections [2]. Furthermore, malnutrition, defined here as undernutrition, is still a heavy burden in these regions where HIV-infected children live [3]. In Western Africa, 18.7% of the general population was estimated to be undernourished in 2020, and 28.8% were severely food insecure. The prevalence of stunting (Height-for-age Z-score<-2 SD) and wasting (Weight-for-Height Z-score<-2SD) in children less than five years of age were respectively 30.9 and 6.9% [4].

Due to their lifelong infection, children living with HIV acquired perinatally are at high risk of co-morbidities including malnutrition [5, 6]. Impaired nutritional status can also exacerbate their immunodeficiency and lead to advanced HIV status [7, 8]. Therefore, among children with severe acute malnutrition (Weight-for-Height Z-score <-3SD), those infected with HIV have a three-fold increased risk of mortality compared to those not HIV-infected [9, 10]. Initiation of ART has been shown to have a positive impact on growth, with significant catch-up growth during the first years of ART, especially in children initiated early, before the age of two years old [11, 12]. However, high rates of malnutrition are found in children receiving long-term ART, illustrating the need for greater consideration of the nutrition component in pediatric HIV care [13, 14].

Currently, the World Health Organization (WHO) recommends that children living with HIV increase their energetic needs by 10% if asymptomatic, by 20–30% during symptomatic phases, and by 50–100% in the case of severe acute malnutrition [15]. However, these guidelines are mainly based on studies conducted in adults, with a low level of evidence in children. Few interventional studies have been conducted specifically in children living with HIV. Two Cochrane reviews had identified eight and three clinical trials on micronutrients and macronutrients supplementation respectively [16, 17], mainly conducted in the early 2000s, prior to global access to ART. In addition, six cohort studies on macronutrients supplementation were identified [18]. Despite the numerous studies conducted on malnutrition in children under five years of age, specific questions remain about nutritional care for children living with HIV [19, 20].

As part of the IeDEA (International Epidemiology Databases to Evaluate AIDS) pediatric cohort, our objective was to assess the longitudinal effect of a 6-month nutritional intervention in children living HIV followed in the IeDEA West African cohort.

## Methods

### Study setting

The study, named WADANUT, was conducted in five pediatric HIV clinics in three West African countries (National University Hospital Center [CNHU] Cotonou, Benin; CHU Tokoin, Lomé, Togo; CHU Cocody, Centre de Prise en charge et de Formation [CePreF] and CHU Yopougon, Abidjan, Côte d’Ivoire). These clinics are part of the IeDEA pWADA (pediatric West African Database on AIDS) collaboration, which is an international research consortium established in 2006 to collect data on HIV and AIDS [21].

### Study design, population and conduct

Between September 2014 and January 2016, a non-comparative longitudinal interventional study consisting of 6 months of nutritional support to children living with HIV, based on their degree of weight deficiency, was held. All children with a confirmed HIV diagnosis, under 10 years of age followed in participating clinics were systematically screened for weight deficiency for an inclusion period of 3 to 6 months, depending on the centers. After screening, children and their caregivers were proposed for their participation in WADANUT and then included if their formal consent was obtained.

Two indicators of malnutrition, wasting and underweight, were combined to define three degrees of weight deficiency: severe, moderate or not. Wasting (also known as acute malnutrition) was defined by Weight-for-Height Z-score (WHZ) for children under five years of age and by Body-Mass-Index-for-age Z-score (BAZ) for children aged five years or older. Underweight was defined by Weight-for-age Z-score (WAZ). Severe weight deficiency was then defined by at least one Z-score (WHZ/BAZ or WAZ) less than -3 Standard Deviations (SD); moderate weight deficiency by at least one Z-score between -3 and -2 SD, both Z-scores being equal to or higher than -3SD; and no weight deficiency by both Z-scores being equal to or higher than -2 SD.

Children received specific nutritional support based on these three categories of malnutrition, in line with the WHO nutritional guidelines for HIV-infected children [15]. First, those who were not weight deficient, pediatricians offered nutritional counseling messages for them and their caregivers. Each participating center adopted its own counseling strategy using, for example, UNICEF pictures and brochures. Second, those who were moderately weight deficient received locally produced fortified blended flours (FBF): Cereso in Benin, Nutrisoy in Togo and CSB+ in Côte d’Ivoire. Children were prescribed a specific amount of product according to their age: 85, 75 and 55 Kcal/kg/day for children aged 0-2 years, 2-5 years and 5-10 years respectively. Finally, those who were severely weight deficient were all prescribed Ready-To-Use Therapeutic Food (RUTF), using Plumpy Nut. The supplemental energy requirements were 220 and 100 Kcal/kg/day for children aged 0-5 years and 5-10 years respectively. Children suffering from severe acute malnutrition requiring hospitalization or with edematous symptoms were not included and were referred to nutritional rehabilitation units.

The children included had a first follow-up visit two weeks after the start of the intervention as a control, providing initial feedback, and were then followed up monthly for 6 months. At each visit, weight deficiency was assessed and the amount of nutritional supplements was adjusted if needed. They were provided with the required nutritional supplements based on their degree of weight deficiency for one month, or less if they were able to collect the supplements at the clinic sooner.

This study adhered to CONSORT guidelines and was retrospectively registered (PACTR202001816232398, 06/01/2020).

### Data collection

Weight and height were collected at each visit by trained health care professionals, following standard procedures [22], using a SECA mechanical scale, a recumbent length scale for children under two years of age and a vertical height scale for children over two years of age. Anthropometric indicators expressed in Z-scores were calculated according to WHO growth standards [23, 24], using reference tables. There were recalculated during the analysis with the WHO Anthro and WHO Anthroplus softwares. In addition to wasting and underweight, stunting, defined as Height-for-Age Z-score (HAZ) was also documented.

At inclusion, data on socio-demographic characteristics (age, sex, primary caregiver, orphanhood) were collected. Other data were collected at inclusion and during follow-up, such as clinical and immunological status (CD4 count, viral load and WHO stage at last measurement, opportunistic infections in the last six months/since last visit), and treatment (ART regimen and cotrimoxazole prophylaxis). During monthly follow-up, children and their caregiver were also asked about adherence to the nutritional product (whether or not it was fully consumed, if not what the reasons were).

In addition, to measure whether receiving a nutritional supplement and counselling would have an impact on usual eating habits, Dietary Diversity Questionnaires (DDQs) with a 24h-recall were conducted at the inclusion and end of nutritional supplementation. For children over two years of age, the DDQ consisted of sixteen food categories. After being described separately, these categories were aggregated into nine groups to obtain the Women’s Dietary Diversity Score (WDDS) [25]. For children under two years of age, the DDQ was composed of seven categories, and the minimum dietary diversity score (MDDS) was defined as having answered yes to at least four categories [26].

Data were collected with paper-based questionnaires and then entered electronically in each country with a standardized and anonymized database. These databases were then sent to France for centralization and analyses.

### Statistical analyses

Characteristics at inclusion were compared by country and by intervention group using Chi-square and Fisher’s exact tests for categorical variables and Student and Wilcoxon tests for quantitative variables. The characteristics of children attending study sites during the inclusion period were compared according to their inclusion in the current study. Dietary diversity scores were compared for each child at the first and last visit using paired Wilcoxon, signed-rank and analysis of variance tests. Recovery from malnutrition was defined as a WAZ and WHZ/BAZ Z-scores greater than or equal to -2 SD at 6 months or at the last visit if it occurred before 6 months. The recovery rate was compared according to the characteristics at inclusion using Chi-square and Fisher’s exact tests.

### Ethics approval and consent to participate

The IeDEA consortium has received formal approvals from the local Institutional Review Boards and U.S. National Institutes of Health to collect data prospectively at each center since 1998. This study has obtained specific approval from the local Institutional Review Board of each participating countries: in Côte d’Ivoire, Comité National d'Ethique des Sciences de la Vie et de la Santé (CNESVS), Ministry of Health and Public Hygiene (IRB 00009111); in Benin: Comité National d'Ethique pour la Recherche en Santé (CNERS), Ministry of Health (IRB 00006860); in Togo: Comité de Bioéthique pour la Recherche en Santé (CBRS), Ministry of Health (IRB 00009547). Individual informed consent was obtained from the primary caregiver (parents or legal representative) for each participant (written approval of the primary caregiver as well as written assent for children aged 7 years or older).

## Results

### General characteristics of the study population

During the inclusion period, 870 eligible children attended the participating centers. Of them, 409 (47%) children were included, 83 (20%) were subsequently excluded of the analysis because of missing information at inclusion (28) and follow-up (45). Children attending the study sites during the inclusion period but who were not enrolled in the study were younger compared to those enrolled (% age 0-2 years: 19 vs 8%, *p*<0.001), more frequently followed in Côte d’Ivoire sites (68% vs 48%), and more often severely immunodeficient (23 vs 17%) (Supplemental Digital Content 1). In addition, these children were not systematically screened for weight and height, resulting in a high rate of missing data (from 24% for underweight to 36% for stunting).

The baseline characteristics of the 326 children included were the following: 48% were from Côte d’Ivoire, 28% from Benin and 24% from Togo; their median age was 6.5 years (interquartile range [IQR] 3.8-8.2), 48% were females, and 28% were orphans of at least one parent. There were no differences by country on these socio-demographic characteristics, except for the primary caregiver who was one of the parents in 93% of the cases in Togo versus 86% in Benin and 75% in Côte d’Ivoire (*p*=0.001) (Table 1).

**Table 1:** Characteristics of the study population at inclusion in the WADANUT study, 2014-2016, *N*=326

Inclusion characteristics	Total ***N***=326		Benin ***N***=91		Côte d’Ivoire ***N***=158		Togo ***N***=77		***p***-value*
	N	%	N	%	N	%	N	%	
**Socio-demographic data**									
**Age at inclusion**									0.415
0-2yrs	27	8.3	11	12.1	13	8.2	3	3.9	
2-5yrs	84	25.8	24	26.4	39	24.7	21	27.3	
5-10yrs	215	66.0	56	61.5	106	67.1	53	68.8	
**Sex**									0.593
Males	170	52.1	46	50.5	87	55.1	38	49.4	
Females	156	47.9	45	49.5	71	44.9	39	50.6	
**Primary caregiver**									0.001
Father, mother	269	82.5	79	86.8	118	74.7	72	93.5	
Other family member	47	14.4	12	13.2	31	19.6	4	5.2	
Other (institute or missing)	10	3.1	0	0.0	9	5.7	1	1.3	
**Orphanhood**									0.570
Non orphan	236	72.4	66	72.5	111	70.3	59	76.6	
One parent	84	25.8	22	24.2	44	27.8	18	23.4	
Two parents	6	1.8	3	3.3	3	1.9	0	0.0	
**Treatments**									
**On antiretroviral therapy**	298	91.4	79	86.8	142	89.9	77	100.0	0.001
Based on PIs	61	20.5	18	22.8	26	18.3	17	22.1	0.294
Based on NRTIs	228	76.5	61	77.2	111	78.2	56	72.7	
Missing data	9	3.0	0	0.0	5	3.5	4	5.2	
**Duration on ART**									0.018
0-2 yrs	120	40.3	37	40.7	65	45.8	18	23.4	
2-5 yrs	105	35.2	25	27.5	44	31.0	36	46.8	
5-10yrs	71	23.8	17	18.7	33	23.2	21	27.3	
Missing data	2	0.7	0	0.0	0	0.0	2	2.6	
**On Cotrimoxazole prophylaxis**	242	74.2	72	79.1	146	92.4	77	100.0	<0.001
**Clinical status and co-morbidities**									
**Immunodeficiency for age**^a^									
No	176	54.0	36	39.6	97	61.4	43	55.8	<0.001
Moderate	39	12.0	6	6.6	25	15.8	8	10.4	
Severe	55	16.9	16	17.6	33	20.9	6	7.8	
Missing data	56	17.2	33	36.3	3	1.9	20	26.0	
**WHO stage**									
I-II	214	65.6	79	86.8	61	38.6	74	96.1	<0.001
III-IV	86	26.4	11	12.1	73	46.2	2	2.6	
Missing data	26	8.0	1	1.1	24	15.2	1	1.3	
**Viral load**									<0.001
<500	52	16.0	37	40.7	16	10.1	0	0.0	
>500	78	23.9	36	39.6	41	25.9	0	0.0	
Missing data	196	60.1	18	19.8	101	63.9	77	100.0	
**Opportunistic infections** **during the last 6 months**									
Tuberculosis	8	2.5	2	2.2	6	3.8	0	0.0	0.262
Pneumonia	23	7.1	10	11.0	8	5.1	5	6.5	0.208
Malaria	47	14.4	6	6.6	9	5.7	32	41.6	<0.001
Diarrhoea	17	5.2	4	4.4	9	5.7	4	5.2	0.948
Other (?)	17	5.2	4	4.4	15	9.5	34	44.2	<0.001
**Malnutrition**^b^									
**Underweight (Weight-for-age)**									0.128
No	245	75.2	65	71.4	115	72.8	65	84.4	
Moderate	55	16.9	15	16.5	30	19.0	10	13.0	
Severe	26	8.0	11	12.1	13	8.2	2	2.6	
**Wasting (Weight-for-Height, BMI for age)**									0.170
No	292	89.6	82	90.1	135	85.4	75	97.4	
Moderate	25	7.7	7	7.7	17	10.8	1	1.3	
Severe	9	2.8	2	2.2	6	3.8	1	1.3	
**Stunting (Height-for-age)**									0.376
No	232	71.2	62	68.1	118	74.7	52	67.5	
Moderate	62	19.0	16	17.6	28	17.7	18	23.4	
Severe	32	9.8	13	14.3	12	7.6	7	9.1	

*ART* antiretroviral therapy, *PIs* Protease Inhibitors, *NRTIs* Nucleoside Reverse Transcriptase Inhibitors, *WHO* World Health Organization

* Chi-square or Fisher’s exact test

^a^ WHO guidelines 2006

^b^ Severe malnutrition: Z-score<-3SD, moderate malnutrition: Z-score=[-3;-2[ SD

ART coverage at inclusion ranged from 87% in Benin to 100% in Togo (*p*=0.006), with overall 77% of ART-treated children on a regimen based on NRTIs (Nucleoside Reverse Transcriptase Inhibitors). The median duration of ART was 2.6 years on ART (IQR 1.0-4.9). Immunological and clinical status at inclusion differed between countries, with the lowest rates of severe immunodeficiency (8%) and advanced WHO stage (3% for stage 3 or 4) being reported in Togo and the highest rates in Côte d’Ivoire (21 and 46% respectively, *p*<0.001). Viral load was not routinely collected in Benin and Togo resulting in a high rate of missing data (60%). In the previous six months, 2, 7 and 5% of cases of tuberculosis, pneumonia and diarrhea were reported, respectively. Malaria was reported in 14% of cases, with the highest rate observed in Togo (42%) (Table 1). The prevalence of malnutrition at inclusion was similar across countries, with 25% underweight, 10% wasting and 29% stunted overall (Table 1).

### Characteristics by group of nutritional support at the start of the intervention and catch-up growth

**Fig. 1:**
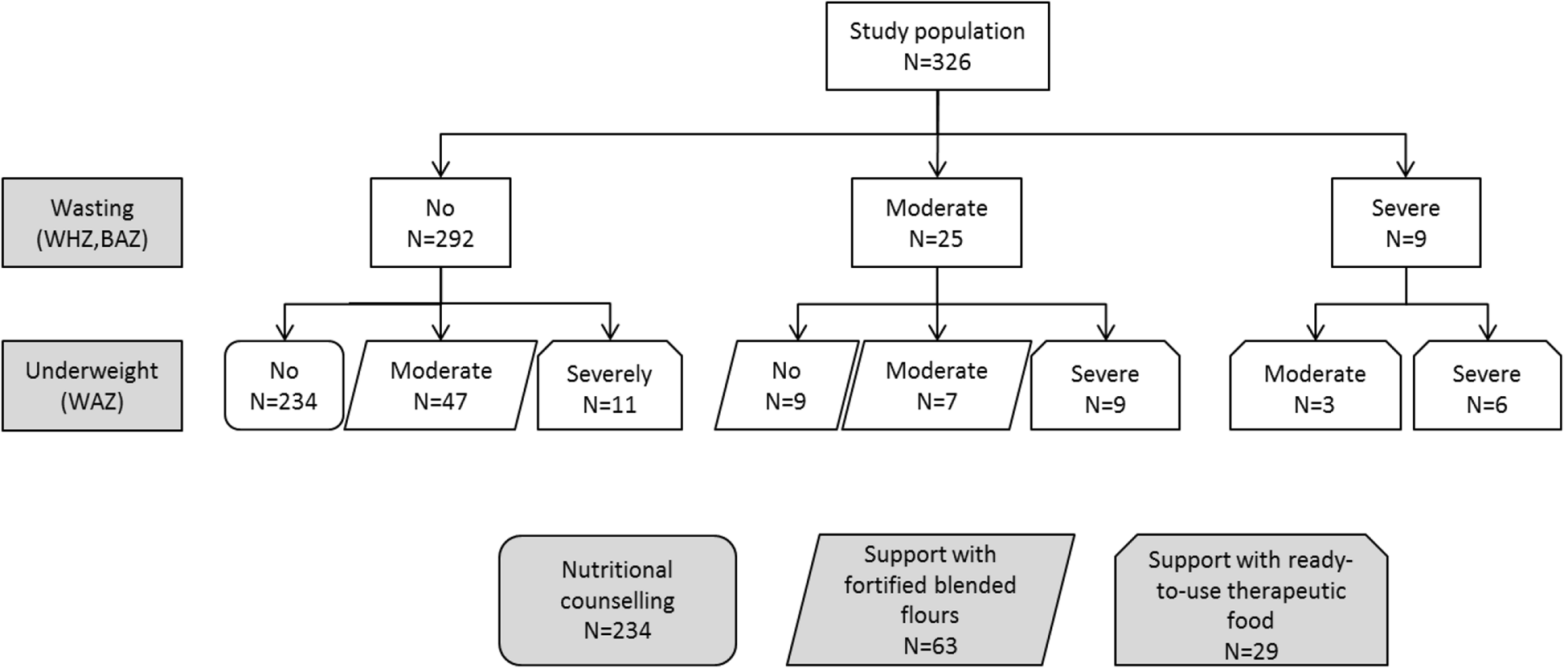
Flow-chart of the interventional study, Wadanut, 2014-2016

At inclusion, the nutritional intervention was proposed to three exclusive groups: 234 (72%) children were considered as not weight deficient and received nutritional counselling (“Counselling” group), 63 (19%) were moderately malnourished and were supplemented with fortified blended flours (“FBF” group), and 29 (9%) were severely malnourished and were supplemented with RUTF (“RUTF” group) (Fig. 1).

At inclusion, children were equally distributed by sex (*p*=0.715) and country (0.109) in the three intervention groups. Children under two years of age were the most severely malnourished, representing 38% of all children in the RUTF group vs. 5% in the other two groups (*p*<0.001). The proportion of severely immmunodeficient children was higher in the FBF group (22%) and the RUTF group (41%) than in the Counselling group (12%) (*p*-value<0.001). The prevalence of stunting was also higher, with respectively 62 and 76% of children with stunting in the FBF RUTF groups, compared to 14% in the Counselling group (*p*-value<0.001) (Table 2). The three intervention groups did not differ according to other socio-economic and treatment characteristics (data not shown).

**Table 2:** Description by nutritional support: comparison of the main characteristics at inclusion and follow-up data, WADANUT study, 2014-2016, *N*=326

Characteristics by nutritional support	Counseling ***N***=234		FBF ***N***=63		RUTF ***N***=29		
At inclusion	N	%	N	%	N	%	***p***-value*
**Age groups**							<0.001
0-2 years	13	5.6	3	4.8	11	37.9	
2-10 years	221	94.4	60	95.2	18	62.1	
**Sex**							0.715
Girls	109	46.6	33	52.4	14	48.3	
Boys	125	53.4	30	47.6	15	51.7	
**Country**							0.109
Benin	64	27.4	16	25.4	11	37.9	
Côte d’Ivoire	107	45.7	35	55.6	16	55.2	
Togo	63	26.9	12	19.0	2	6.9	
**Immunodeficiency by age**^a^							<0.001
No	133	56.8	36	57.1	7	24.1	
Moderate	32	13.7	2	3.2	5	17.2	
Severe	29	12.4	14	22.2	12	41.4	
Missing	40	17.1	11	17.5	5	17.2	
**Stunting**							<0.001
Yes (HAZ<-2 SD)	33	14.1	39	61.9	22	75.9	
No (HAZ≥-2 SD)	201	85.9	24	38.1	7	24.1	
**Follow-up**							
Median time of follow-up (IQR)	4.9 (3.0-5.7)		4.6 (2.8-6.1)		4.3 (3.1-5.4)		
Mean weight gain (kg) at last visit (sd)	0.97 (1.42)		1.38 (1.66)		1.87 (1.65)		
Mean WAZ gain at last visit (sd)	0.07 (0.45)		0.38 (0.77)		0.97 (1.24)		
Mean WHZ/BAZ gain at last visit (sd)	0.07 (0.74)		0.37 (0.72)		1.15 (1.69)		

*WAZ* Weight-for-Age Z-score, *WHZ/BAZ* Weight-for-Height/BMI-for-Age Z-score, *HAZ* Height-for-Age Z-score, *FBF* Fortified Blended Flours group, *RUTF* Ready-to-Use Therapeutic Food group

* Chi-square or Fisher’s exact test

^a^ WHO guidelines 2006

The median time of study follow-up differed between intervention groups, from 4.3 months (IQR 3.1-5.4) for the RUTF group to 4.9 months (IQR 3.0-5.7) for the Counselling group. Adherence to nutritional products was not systematically reported during follow-up and therefore could not be quantified, but some qualitative reasons to non-adherence were raised. For example, some children did not like the porridge made with FBF, others did not like Plumpy-Nut either. They also expressed their tiredness at taking nutritional supplements for a long period of time and preferred to eat family dishes.

At the end of follow-up, the mean weight gain per patient in the FBF group was 1.4 kg (sd 1.7), corresponding to a mean increase of 0.4 SD (sd 0.7) for both WAZ and WHZ/BAZ. In the RUTF group, the mean weight gain per patient was 1.9 kg (sd 1.6), with a mean increase of 1.0 SD (sd 1.2) for WAZ and 1.1 (sd 1.7) for WHZ/BAZ (Table 2).

After 6 months of follow-up (or at the last visit if earlier), 31/63 (49%) of the children in the FBF group recovered from moderate malnutrition. The rate of recovery from malnutrition for the FBF group did not differ by age, sex, immunodeficiency by age and ART duration, but was higher in Côte d’Ivoire where 74% of recoveries occurred (*p*-value=0.013). Also, 75% of the children who did not recover were stunted, compared to 48% of the children who recovered (*p*-value=0.030). In the RUTF group, 9/29 (31%) fully recovered from their severe malnutrition, and 7/29 (24%) partially recovered, remaining moderately malnourished at the end of the study. The rate of complete recovery from malnutrition did not differ by age, country, immunodeficiency by age and ART duration. Trends towards greater recovery in boys compared to girls were observed (7/15 vs 2/14) but the sample size was too small to conclude a significant difference (*p*-value=0.109). Similarly, stunting seemed to alter the recovery rate (Table 3). Also, 11/234 (5%) of the initially non-malnourished children developed moderate malnutrition during follow-up but no differences were observed based on characteristics at inclusion (data not shown).

**Table 3:** Weigth gains and catch-up growth at the last visit or at 6 months of follow-up for malnourished children, compared with main characteristics, WADANUT study, 2014-2016, *N*=92

	FBF							RUTF						
	WHZ/BAZ gains, mean Z-score (sd)	WAZ gains, mean Z-score (sd)	Not malnourished		Malnourished		*p*-value*	WHZ/BAZ gains, mean Z-score (sd)	WAZ gains, mean Z-score (sd)	Not malnourished		Malnourished		*p*-value
			N	%	N	%				N	%	N	%	
Overall	0.37 (0.72)	0.38 (0.77)	31.0	49.2	32	50.8		1.15 (1.69)	0.97 (1.24)	9	31.0	20	69.0	
**Age groups**							0.613							0.772
0-2 years	0.17 (2.13)	0.29 (1.85)	2	6.5	1	3.1		1.69 (2.22)	1.46 (1.65)	3	33.3	8	40.0	
2-10 years	0.38 (0.62)	0.38 (0.70)	29	93.5	31	96.9		0.82 (1.22)	0.65 (0.78)	6	66.7	12	60.0	
**Sex**							0.701							0.109
Girls	0.45 (0.67)	0.27 (0.59)	17	54.8	16	50.0		0.58 (1.30)	0.52 (1.15)	2	22.2	12	60.0	
Boys	0.29 (0.76)	0.49 (0.92)	14	45.2	16	50.0		1.67 (1.88)	1.36 (1.22)	7	77.8	8	40.0	
**Country**							0.013							0.712
Benin	0.21 (0.76)	0.33 (0.62)	5	16.1	11	34.4		1.09 (1.12)	0.94 (0.84)	3	33.3	8	40.0	
Ivory Coast	0.50 (0.76)	0.35 (0.56)	23	74.2	12	37.5		1.18 (1.99)	0.99 (1.44)	6	66.7	10	50.0	
Togo	0.18 (0.41)	0.51 (1.29)	3	9.7	9	28.1		1.25 (2.74)	0.93 (2.02)	0	0.0	2	10.0	
**Immunodeficiency by age**^a^							0.423							0.599
No	0.42 (0.59)	0.42 (0.82)	20	64.5	16	50.0		0.78 (1.27)	0.61 (0.81)	3	33.3	4	20.0	
Moderate	-0.88 (1.37)	-1.61 (-)	1	3.2	1	3.1		1.30 (2.20)	1.19 (1.62)	2	22.2	3	15.0	
Severe	0.42 (0.59)	0.58 (0.69)	7	22.6	7	21.9		1.40 (1.91)	1.14 (1.36)	3	33.3	10	50.0	
Missing	0.16 (0.33)	0.19 (0.33)	3	9.7	8	25.0		0.90 (1.50)	0.76 (1.18)	2	22.2	3	15.0	
**Stunting**							0.030							0.158
Yes (HAZ<-2 SD)	0.17 (0.63)	0.34 (0.89)	15	48.4	24	75.0		1.02 (1.88)	1.01 (1.40)	5	55.6	17	85.0	
No (HAZ≥-2 SD)	0.69 (0.75)	0.43 (0.51)	16	51.6	8	25.0		1.53 (0.89)	0.84 (0.58)	4	44.4	3	15.0	

*WAZ* Weight-for-Age Z-score, *WHZ/BAZ* Weight-for-Height/BMI-for-Age Z-score, *HAZ* Height-for-Age Z-score

Fortified Blended Flours group, *RUTF* Ready-to-Use Therapeutic Food group

Not malnourished = WAZ and WHZ/BAZ Z-scores ≥-2 SD

* Chi-square or Fisher’s exact test

^a^ WHO guidelines 2006

### Dietary diversity and nutritional habits

Among children under two years of age at inclusion, 24/27 had dietary diversity data at the first visit, and 14 at the last visit. Of the seven food groups asked for infants, the cereals group was cited by all, and the dairy products, flesh foods and other fruits and vegetables groups by more than half. The mean scores at inclusion and at the last visit were respectively 3.7 (SD 1.3) and 4.1 (SD 0.9) (p-value signed rank=0.019). Overall, 11/24 (46%) reached the MDDS on the first visit, and 10/14 (71%) on the last visit. The score at the first visit was lower in Benin (mean 3 [SD 0.6], 18% having a MDDS) than in Côte d’Ivoire and Togo (overall: mean 4.3 [sd 1.4], 69% having a MDDS, p-value analysis of variance 0.034).

**Fig. 2:**
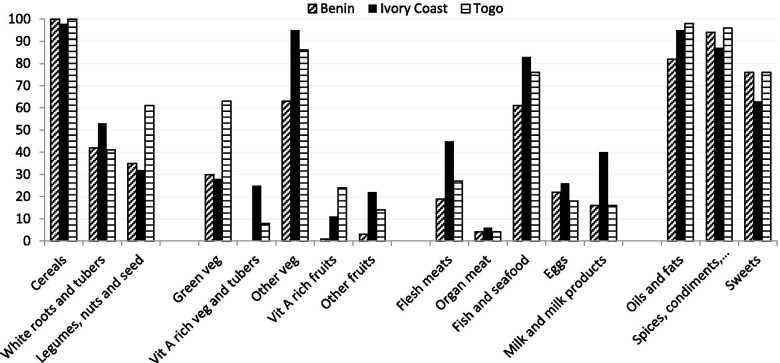
Dietary diversity by country, at first visit, for children aged more than two years at inclusion, frequencies of consumption,WADANUT, 2014-2016

Among children over 2 years of age at inclusion, 256 completed the dietary diversity questionnaire at the first visit, and 166 of them also at the last visit. At inclusion, 100% of children in Côte d’Ivoire and Togo reported having breakfast in the morning and 83% in Benin. Overall, in Benin, Côte d’Ivoire and Togo respectively, 79%, 96% and 79% of children took three meals, and 78%, 79% and 81% took at least two snacks per day. Results were similar at the end of the study (data not shown). Frequencies of consumption at the first visit, for all sixteen groups, are displayed in Fig. 2. Trends were similar on the last visit. All children reported cereals, and more than half reported oils and fats (92%), spices, condiments and beverages (91%), fish and seafood (75%) and other vegetables (84%) categories. Sweets were also reported by more than 60% of the population. The least reported food groups were organ meat (5%), vitamin A rich fruits (11%), vitamin A rich vegetables and tubers (14%) and others fruits (14%). Food consumption differed by country, with for example the highest consumption of flesh meats (45%), fish and seafood (83%), white roots and tubers (53%), and milk products (40%) in Côte d’Ivoire. Legumes, nuts and seed (61%) and green vegetables (63%) were reported more often in Togo. The consumption of Vitamin A fruits and vegetables was barely reported in Benin. Overall, the mean WDDS was 4.3 (sd 1.2) at the first visit, with no significant difference at the last visit (paired p-value=0.907), with 62% of children consuming less than 5 out of 9 different food groups. This score differed by country, with a lower WDDS in Benin than in Côte d’Ivoire and Togo (first visit: 3.5 [sd 0.9] vs 4.6 [sd 1.1], p-value<0.001). In Benin, 89% of children consumed less than 5 different food groups, compared to 52% and 47% respectively in Côte d’Ivoire and Togo (*p*-value<0.001). The WDDS did not differ by intervention group (*p*-value=0.767) or by sex, age and severity of malnutrition at inclusion (Table 4).

**Table 4:** Mean Women’s Dietary Diversity Score for children over 2 years of age, by country and nutritional support group, at first and last visit. WADANUT study, 2014-2016

Variables	At the first visit (***N***=256)				At the last visit (***N***=166)			
	N	Mean	SD	***p***-value*	N	Mean	SD	***p***-value
Overall		4.3	1.2			4.2	1.1	
**Country**				<0.001				<0.001
Benin	79	3.5	0.9		61	3.6	0.8	
Côte d’Ivoire	126	4.6	1.1		75	4.6	1.1	
Togo	51	4.6	1.2		30	4.5	1.1	
**Intervention group**				0.767				0.489
Counselling	186	4.3	1.2		117	4.2	1.1	
FBF	51	4.3	1.1		36	4.3	1.0	
RUTF	18	4.1	1.2		13	3.9	0.8	

*Student test

## Discussion

Our study provides original longitudinal data on nutritional support for West-African children living with HIV. The 6-month nutritional support provided according to the severity of malnutrition (wasting and underweight) did not greatly help improving their nutritional status. Overall, only 31/63 (49%) and 9/29 (31%) children with moderate and severe malnutrition respectively have fully recovered from their weigh deficiency after 6 months. However, the rate of recovery from malnutrition seemed to be better for those who were not stunted at inclusion. This study also provided original data on dietary diversity, which did not change over the course of the intervention, with no differences by sex, age or intervention groups but a lower WDDS in Benin compared to Côte d’Ivoire and Togo.

The literature on nutritional support interventions among children living with HIV is scarce, and few studies have looked at the effect of macronutrient supplementation, especially in West-Africa where malnutrition is prevalent [17, 18]. In Malawi, malnourished children living with HIV received home-based therapy using either RUTF or blended flours, showing that 56% recovered from wasting, with better improvements for those who received RUTF. However, this study was conducted only among non-ART treated children [27]. In Tanzania, a cross-sectional study among ART-treated children showed that RUTF use for at least four months was associated with low rates of underweight, wasting, and stunting [28]. More recently, a study in Mali assessing the use of RUTF in ART-treated children showed a 79 and 60% probability of recovery from malnutrition after 6 months for those with moderate and severe acute malnutrition respectively. Similarly to our study, concurrent stunting was associated with a lower probability to recover from acute malnutrition [29].

Comparable studies on dietary diversity in children living with HIV were very limited. A cross-sectional study in Ethiopia defined good dietary diversity for 48% of children living with HIV [30]. In this study, unlike ours, dietary diversity was associated with the level of malnutrition. In the same country, among children under five years of age, food security was associated with stunting [31]. Low dietary diversity could be explained by factors not recorded here such as food insecurity, poverty, or illiteracy [31], as well as seasonality in rural contexts [32, 33]. The results of our study, conducted only in urban settings, may not have been impacted by this seasonality effect.

Adherence to nutritional products was not optimal in our study. These results were also documented for the use of RUTF in acutely malnourished children and adolescents living with HIV in Senegal [34, 35], with for example reports of disgust feelings, self-stigma and practices of sharing with the households. In Mali, the RUTF consumption was also reported as incomplete for one third of the study population [29].

There are several limitations to our study. First, the recruitment of children was lower than expected, leading to a small sample size and insufficient statistical power to highlight significant associations. This recruitment issue may be explained by a lack of time for healthcare professionals to involve their patients in the study by providing informed consent, or by the refusal of families to come back to the clinic monthly. When comparing characteristics of children recruited or not recruited during the inclusion period, those who were not recruited were generally younger and more severely immunodeficient than the study population, thus the most vulnerable children may not have been recruited for the intervention. In addition, it is likely that the legal caregiver was not always attending the visit to allow for informed consent prior to inclusion. The inclusion rate was the lowest in Côte d’Ivoire, where the study centers had the largest number of patients, as healthcare professionals may have been more overworked and therefore could not offer the intervention to all their patients.

The low efficacy of the intervention could be partly explained by inconsistent follow-up, as monthly visits and the supply of nutritional products may be too constraining for patients and healthcare professionals. The distribution of the products and the nutritional assessment in the clinics may not have been entirely appropriate. Also, the nutritional products chosen for this protocol may have been inadequate for this study population. Although RUTF have been shown to be effective for malnourished children and adults [36, 37], few studies have used these products in children living with HIV older than five years of age [20]. Information about adherence to the nutritional products were not collected systematically during the intervention and relied on self-reporting from the caregivers, which may not have been the most appropriate way to measure adherence, with a risk of desirability bias.

Furthermore, information on food insecurity and biological data could have been useful for further analysis, but were not recorded due to time and funding restraints. Food insecurity has been shown to be a contributing factor to low levels of macro and micronutrients intakes [38] and the HIV-affected population may be more food insecure than the general population [39, 40]. Biological data on lipid profiles and glycaemia could also have been useful in understanding the effect of these high-energy nutritional products on metabolism [37]. HIV-related data, such as CD4 counts and viral load, were not consistently performed over the intervention period and thus the effect of nutritional supplementation on HIV outcomes could not have been assessed.

The use of a threshold of -2 SD to consider recovery from malnutrition may not be seen as a marker of sustainable recovery. Long-term recovery may have been defined using additional anthropometric measurements, such as three months after the end of the intervention if resources were available.

Finally, the intervention was not comparative, the use of a control group that would not have benefited from the intervention in the same time than the intervention group was considered unethical by the research team. A stepped-wedge design, where all participants eventually receive the intervention but at different time periods could have been considered.

Nevertheless, our study has helped to better document the nutritional outcomes of children living with HIV, which remain a neglected topic in this vulnerable population. The fact that our study was multi-country, using local nutritional products allowed us assessing the effectiveness of the intervention in different geographic settings. In addition, many children were over five years of age at inclusion, while pediatric nutritional interventions often focus on the first 1000 days of life. Our study also provides original data on dietary diversity, and emphasizes the need for further work in this field to allow comparisons across settings and populations. The use of two anthropometric indicators (WAZ and WHZ/BAZ) allowed taking into account a broader population of children with weight deficiency.

Malnutrition is a burden for children living with HIV in West Africa [14] and we strongly feel that the first year of ART initiation could be considered a critical time to provide nutritional support. Nutritional interventions that would be suitable for children living with HIV and implemented in a sustainable way still need to be further developed and assessed [41, 42]. Innovative ways of delivering nutritional products, such as using community workers [43] could be tested in resource-limited settings where transport to the clinic can be costly and time-consuming for patients and their families. Other than the use of nutritional products, cash-transfer methods [44, 45] as well as nutritional education for mothers [46] could be additional approaches to improve nutritional status of children living with HIV. Interventions focusing on dietary diversity and food security must also be developed to improve their nutritional status [47]. Qualitative studies documenting in more detail the potential barriers to nutritional care [48] for caregivers and their children could also be useful. Healthcare professionals also need better support in this process [49]. Combined interventional studies with ART and nutritional support that provide long-term benefits should be tailored, as it has been shown in several studies in adults [50].

## Conclusions

There is still much to be done to find nutritional care approaches that are appropriate for children living with HIV, involving patients and their families, as well as healthcare professionals, with the support of the community and stakeholders. The results of our intervention study, although reporting limited efficiency, highlight the difficulty of optimizing nutritional care for children living with HIV in West Africa under real-life conditions. While access to food is expected to decrease with the ongoing COVID pandemic, this highly vulnerable population should not be forgotten in supportive nutrition programs [51].

## Supplementary Information

**Additional file 1: Supplemental Digital Content 1.** Baseline characteristics of the 870 eligible children seen during the inclusion period according to their inclusion status in the current study.

## Data Availability

The datasets generated and/or analysed during the current study are not publicly available, as data ownership remains with the participating sites. Each site has approval from its own local Institutional Review Board to collect routine data on patients and to transfer those data anonymously to the IeDEA West Africa collaboration. Reasonable request for access to data can be addressed to the corresponding author.

## Abbreviations

ART: Antiretroviral Therapy
DDQ: Dietary Diversity Questionnaire
DDS: Dietary Diversity Score
FBF: Fortified Blended Flours
IeDEA: International Epidemiology Databases to Evaluate AIDS
HAZ: Height-for-Age Z-score
MDDS: Minimum Dietary Diversity Score
MTCT: Mother-to-Child Tranmission
NRTIs: Nucleoside Reverse Transcriptase Inhibitors
PIs: Protease Inhibitors
PMTCT: Prevention from mother-to-child transmission
RUTF: Ready-to-Use Therapeutic Food group
WAZ: Weight-for-Age Z-score
WHZ/BAZ: Weight-for-Height/BMI-for-Age Z-score
WDDS: Women’s Dietary Diversity Score
