# Supplementary material for: Assessment of dietary diversity and nutritional support for children living with HIV in the IeDEA pediatric West African cohort: a non-comparative, feasibility study
Source: BMC Nutr. 2021 Dec 14;7:83. doi: 10.1186/s40795-021-00486-4 (PMC8670202; doi:10.1186/s40795-021-00486-4)
Supplement: Supplementary file 1 — Additional file 1: Supplemental Digital Content 1. Baseline characteristics of the 870 eligible children seen during the inclusion period according to their inclusion status in the current study. [file 40795_2021_486_MOESM1_ESM.docx]

Supplemental Digital Content 1: Baseline characteristics of the 870 eligible children seen during the inclusion period according to their inclusion status in the current study.

| **Inclusion characteristics (N, %)** | | **Total** | | | | **p-value*** |
| --- | --- | --- | --- | --- | --- | --- |
|  |  | **Included**  **(N=326)** | | **Excluded**  **(N=544)** | |  |
| **Age at inclusion** | |  |  |  |  | <0.001 |
| 0-2yrs | 27 | 8.3 | 102 | 18.8 |  |  |
| 2-5yrs | 84 | 25.8 | 104 | 19.1 |  |  |
| 5-10yrs | 215 | 66.0 | 338 | 62.1 |  |  |
| **Sex** | |  |  |  |  | 0.540 |
| Males | 170 | 52.1 | 272 | 50.0 |  |  |
| Females | 156 | 47.9 | 272 | 50.0 |  |  |
| **Country** | |  |  |  |  | <0.001 |
| Benin | 91 | 27.9 | 49 | 9.0 |  |  |
| Côte d'Ivoire | 158 | 48.5 | 370 | 68.0 |  |  |
| Togo | 77 | 23.6 | 125 | 23.0 |  |  |
| **On antiretroviral therapy** | | 298 | 91.4 | 484.0 | 89.0 | 0.086 |
| **Duration on ART** | |  |  |  |  | <0.001/0.312 |
| 0-2 yrs | 120 | 40.3 | 203 | 41.9 |  |  |
| 2-5 yrs | 105 | 35.2 | 151 | 31.2 |  |  |
| 5-10yrs | 71 | 23.8 | 90 | 18.6 |  |  |
| Missing data | 2 | 0.7 | 40 | 8.3 |  |  |
| **Immunodeficiency for age†** | |  |  |  |  |  |
| No | 176 | 54.0 | 75 | 13.8 | <0.001/<0.001 |  |
| Moderate | 39 | 12.0 | 88 | 16.2 |  |  |
| Severe | 55 | 16.9 | 127 | 23.3 |  |  |
| Missing data | 56 | 17.2 | 254 | 46.7 |  |  |
| **Underweight**§ | |  |  |  |  | <0.001/0.729 |
| No | 245 | 75.2 | 311 | 57.2 |  |  |
| Moderate | 55 | 16.9 | 70 | 12.9 |  |  |
| Severe | 26 | 8.0 | 30 | 5.5 |  |  |
| Missing data | 0 | 0.0 | 133 | 24.4 |  |  |
| **Wasting**§ | |  |  |  |  | <0.001/0.619 |
| No | 292 | 89.6 | 304 | 55.9 |  |  |
| Moderate | 25 | 7.7 | 25 | 4.6 |  |  |
| Severe | 9 | 2.8 | 14 | 2.6 |  |  |
| Missing data | 0 | 0.0 | 201 | 36.9 |  |  |
| **Stunting**§ | |  |  |  |  | <0.001/0.249 |
| No | 232 | 71.2 | 265 | 48.7 |  |  |
| Moderate | 62 | 19.0 | 53 | 9.7 |  |  |
| Severe | 32 | 9.8 | 27 | 5.0 |  |  |
| Missing data | | 0 | 0.0 | 199 | 36.6 |  |

(Footnotes SDG1) * Chi-square or Fisher tests, second p-value corresponding to the test without taking into account the missing data category † WHO guidelines 2006, § Severe malnutrition: Z-score<-3SD, moderate malnutrition: Z-score=[-3;-2[ SD.
